# Benefits of Four-Tiered Classification of Exercise-Induced Left Ventricular Hypertrophy in Adolescent Athletes

**DOI:** 10.3390/jcm15176879

**Published:** 2026-09-05

**Authors:** Dora Szabo, Dora Babocsay, Kata Eklics, Istvan Szokodi, Miklos Toth, Pongrac Acs, Attila Cziraki, Zsolt Sarszegi

**Affiliations:** 1 Heart Institute, Medical School, University of Pecs, 7624 Pecs, Hungary; 2 Department of Languages for Biomedical Purposes and Communication, Medical School, University of Pecs, 7624 Pecs, Hungary; 3 Szentagothai Research Centre, University of Pecs, 7624 Pecs, Hungary; 4 Institute of Physiotherapy and Sport Science, Faculty of Health Science, University of Pecs, 7621 Pecs, Hungary; 5 Department of Laboratory Medicine, Semmelweis University, 1089 Budapest, Hungary

**Keywords:** adolescent athletes, exercise-induced left ventricular hypertrophy, four-tiered classification

## Abstract

**Background:** Exercise-induced left ventricular hypertrophy (LVH) has been extensively investigated in adolescent athletes using the conventional two-tiered classification (2TC). However, the four-tiered classification (4TC) allows further differentiation of LVH patterns by incorporating three-dimensional information on left ventricular (LV) geometry. This study aimed to compare LVH assessment using the two classification systems and characterize the reclassification patterns. **Methods:** A total of 121 adolescent athletes (mean age: 15.1 ± 1.6 years) and 114 adult athletes (mean age: 22.9 ± 3.7 years), all competing at the national level, underwent comprehensive echocardiographic and anthropometric evaluations. **Results:** The application of 4TC resulted in redistribution across LV geometric categories compared with conventional 2TC. Twenty-three (19.0%) adolescent and 25 (22%) adult athletes were reclassified, including seven (5.8%) and 17 (15%), respectively, who shifted from normal geometry under the 2TC to different LVH categories under the 4TC. Reclassification was observed across sex and sporting discipline subgroups, although the patterns varied. In runners and triathletes, the combined prevalence of eccentric non-dilated and eccentric dilated LVH was 22.7% using the 4TC versus 4.5% eccentric LVH under the 2TC classification. LVM was strongly associated with the combined contribution of cumulative training duration, lean body mass, and body surface area (r = 0.785, *p* < 0.001), whereas training duration alone was not significantly associated with the LVH parameters. **Conclusions:** The 2TC and 4TC approaches result in different assessments of LV geometry in highly trained athletes, with the 4TC providing additional characterization of LVH patterns by considering the presence or absence of LV dilatation. Reclassification patterns varied according to sex and sporting discipline, highlighting the importance of sport-related characteristics when interpreting exercise-induced LV remodeling in athletes.

## 1. Introduction

Exercise-induced left ventricular adaptation has been widely investigated in adolescent athletes over the past few years. It is clearly defined that the normal values of adult athletes cannot be transferred to children or young adults [1]. Accordingly, recent studies have aimed to establish age- and population-specific reference values for young athletes. Krysztofiak and co-workers examined almost 800 child and adolescent athletes, defining not only the specific age-matched normal values, but also specific normative data of left ventricular mass (LVM) in relation to gender and lean body mass (LBM) [2]. Cavarretta et al. evaluated reference values of left ventricular (LV) structural parameters in a large population of trained adolescents [3]. The importance of population-specific reference values in adolescent athletes extends beyond cardiac morphology. A recent systematic review of normative cardiopulmonary exercise testing (CPET) data in children and adolescents demonstrated considerable variability in normative CPET values according to age, sex, body size and composition, and physical fitness, emphasizing the importance of using population-appropriate reference values for CPET. Kasiak and co-workers also highlighted the limited representation of young athletes in existing normative datasets [4]. Taken together, these findings reinforce the importance of appropriate age-, sex-, body size-, and training-specific reference standards for the evaluation of young athletes.

Normally, left ventricular hypertrophy (LVH) reflects a physiological adaptation to exercise in athletic individuals [5] and conveys an excellent prognosis [6]. The basic principle of LVH evaluation is the estimation of LVM using echocardiography [7]. LVM can increase through LV cavity dilatation, wall thickening, or a combination of these factors [6]. Exercise-induced LV remodeling appears to be a phasic phenomenon, where the increase in LV cavity size may precede or follow the increase in LV wall thickness, based on the type and level of sports activity, finally leading to LVH [7,8,9]. Traditionally, exercise-induced left ventricular hypertrophy has been characterized by a two-tiered classification (2TC). However, this approach has a limited ability to distinguish physiological adaptation from maladaptive or pathological LVH, particularly in adolescent athletes. Furthermore, exercise-induced changes in LV geometry or LVH are influenced by multiple factors, including age, sex, time and intensity of training, cumulative training duration, and sports discipline [8].

The crucial point of differential diagnosis is distinguishing adaptive LVH from maladaptive or pathological changes that may lead to life-threatening arrhythmias or sudden cardiac death (SCD). Finocchiaro et al. investigated the causes and circumstances of SCD in a large cohort of athletes, as determined by post-mortem examination performed by an expert cardiac pathologist. Among all cases, idiopathic left ventricular hypertrophy (LVH) and/or fibrosis accounted for 59 deaths (16%), and among the subpopulation of ≤18 years old, the prevalence of idiopathic LVH/myocardial fibrosis–caused death was 10% [9]. These findings confirm that the current LVH classification is not sensitive enough to detect maladaptive changes.

In the Dallas Heart Study, the new four-tiered classification (4TC) of LVH was examined in a large, population-based cohort. Applying the EDV and concentricity parameters, concentric and eccentric LVH can be subclassified into two subgroups, yielding four different geometric patterns [10]. Garg et al. also examined the potential risk of adverse cardiovascular outcomes in different LVH groups in the general population. They found an increase in heart failure and cardiovascular death in the eccentric and concentric dilated subgroups [11].

Trachsel et al. proved that the four-tiered classification (4TC) discriminated against different forms of exercise-induced left ventricular hypertrophy more than the two-tiered classification in non-elite long-distance runners [12]. Furthermore, 4TC characterization of LVH showed a better correlation between the type and intensity of training, cumulative training hours, and type of LVH. Considering that cumulative training time-dependent changes in LV geometry may be pronounced in young athletes and the growth-related increase in the left ventricular long axis, 4TC seems to be more suitable for assessing LV geometry and LVH in children and adolescent athletes. 4TC assesses LV concentricity, describing the ratio of LV mass to LV end-diastolic volume (LV-EDV), which represents the physiological response in endurance athletes, showing a proportional enlargement of all cavity dimensions while maintaining a normal LV mass/volume ratio [12,13].

### Aim of This Study

Despite growing evidence supporting the use of the four-tier classification (4TC) system in adult populations, its applicability in adolescent athletes remains unclear. Therefore, the objective of this study was to characterize exercise-induced left ventricular adaptation in adolescent athletes. We assessed the left ventricular geometry using 2TC and 4TC. The primary aim of this study was to investigate how the 4TC approach modifies and further refines the classification of LV geometry compared with the conventional 2TC system, with particular emphasis on the characterization and reclassification of different LVH phenotypes. Furthermore, we investigated whether anthropometric parameters and cumulative training duration influenced the type of left ventricular hypertrophy.

The secondary objective was to study the impact of age on the forms of LVH. We selected adult endurance athletes with similar sports disciplines and compared their LV parameters with those of the adolescent athletes. Adult athletes had significantly higher BSA, BMI, and cumulative training time. Therefore, we assumed that this comparison could provide additional information about the effects of anthropometric parameters and cumulative training duration on the types of LVH observed.

## 2. Materials and Methods

The study population was recruited from six sports clubs located in Pécs, Hungary, between September 2021 and December 2022. Adolescent athletes attended primary and secondary schools in the same geographical region; however, recruitment was performed through sports clubs rather than through schools.

A total of 125 adolescent and 115 adult athletes were evaluated in this study. Among the adolescent athletes, two were excluded due to bicuspid aortic valve disease associated with moderate aortic regurgitation, and two additional athletes were excluded due to an atrial septal defect associated with a significant left-to-right shunt. In the adult group, one athlete was excluded because of a Brugada pattern detected on resting electrocardiogram. All five excluded athletes were referred for further cardiological evaluations.

Following these exclusions, the final study population included 121 adolescent athletes (age: 15.1 ± 1.6 years; 96 males) and 114 adult athletes (age: 22.87 ± 3.7 years; 84 males). The sporting disciplines represented in the adolescent and adult groups were long-distance running/triathlon (18.2% vs. 15.8%), basketball (25.6% vs. 49.1%), and soccer (56.2% vs. 35.1%). All participants competed at the national level and were classified as Tier 3 (highly trained/national level) based on the framework described by McKay et al. [14]. According to the AHA/ACC classification of sports, all participants were categorized as Group C, representing sports with a high dynamic component, defined by exercise intensities exceeding 70% of maximal oxygen uptake (VO_2_max), resulting in a substantial increase in cardiac output [15]. However, the static component varied across sporting disciplines (Group I–III.). Adolescent athletes completed 7.5 h of training per week (5 × 1.5 h sessions), with each session comprising 1 h of high-intensity aerobic training and 30 min of low-intensity technical training, whereas adult athletes completed 10.5 h per week (7 × 1.5 h sessions) with the same training structure. The average number of years spent in competitive sports was 6.2 years for adolescent athletes and 10.7 years for adult athletes. A complete physical examination, anthropometric measurements, 12-lead ECG, and echocardiography were performed on all athletes during the season. Echocardiographic examinations were performed at least 16 h after the last training session was completed. Body weight was measured with light clothing using a digital balance scale (Seca, Hamburg, Germany), and height was measured using a wall-mounted stadiometer (Seca, Hamburg, Germany). The body mass index (BMI) was calculated as body mass divided by stature squared (kg/m^2^). The body surface area (BSA) was calculated using the DuBois formula [16]. The Boer Formula was used to calculate the lean body mass (LBM) [17]. All examinations were performed at the Heart Institute of the University of Pécs, Pécs, Hungary, during the study period.

### 2.1. Echocardiographic Measurements

ECG-gated echocardiographic measurements were performed at rest after 10 min of lying down supine. A normal heart rate was detected in all cases between 50 and 70 beats per minute. Two-dimensional (2D), M-mode, Doppler, and tissue Doppler echocardiographic examinations were performed using a high-quality portable echocardiograph (Vivid iq Premium-GE HealthCare, Milwaukee, WI, USA). During the examination, a one-lead ECG was continuously performed. We examined the LV structure (end-systolic and diastolic diameter (LV-ESD and LV-EDD), respectively) and septal/posterior wall thickness (IVS and PWT, respectively) in M-mode from the parasternal long axis view at end-diastole. LV mass (LVM) was calculated using the following formula: *LV mass* = 0.8 × 1.04 × [(*IVS* + *LV-EDD* + *PWT*)3 − *LV-EDD3*] + 0.6 g, indexed to BSA.

LV end-diastolic (LV-EDV) and LV end-systolic volumes (LV-ESV) were calculated using the biplane method of disk summation technique. Volume measurements were based on tracings of the blood-tissue interface in apical four- and two-chamber views.

Left ventricular hypertrophy was defined as LV mass/BSA ≥ 112 g/m^2^ in men and ≥89 g/m^2^ in women [18].

For the 2TC, the relative wall thickness (RWT) was calculated as 2 × *PWT/LV-EDD* (Table 1). A value > 0.42 was considered indicative of concentric geometry [16].

For the 4TC, the left ventricular geometry was specified by LV concentricity (LV mass/LV-EDV2/3 and LVEDV/BSA, as recommended by Khouri et al. [10]. The cut-off values for increased concentricity were ≥9.1 g/mL^2/3^ in men and ≥8.1 g/mL^2/3^ in women, and for increased LVEDV/BSA ≥74 mL/m^2^ in men and ≥68 mL/m^2^ in women.

LV diastolic function and right ventricular structural and functional parameters were also assessed. These measurements were performed according to the latest echocardiographic guidelines; the calculated parameters and indices were defined based on the recommendations of the EACVI and the American Society of Echocardiography [18].

All examinations and measurements were performed by a single investigator. All measurements were repeated by a second cardiologist to rule out any misinterpretation. Intra-and inter-observer variability was assessed using the intraclass correlation coefficient (ICC), a two-way mixed-effects model based on absolute agreement with single measurements (ICC [3, 1]) [19].

### 2.2. Two-Tiered and Four-Tiered Classification of Left Ventricular Hypertrophy

**Figure 1:**
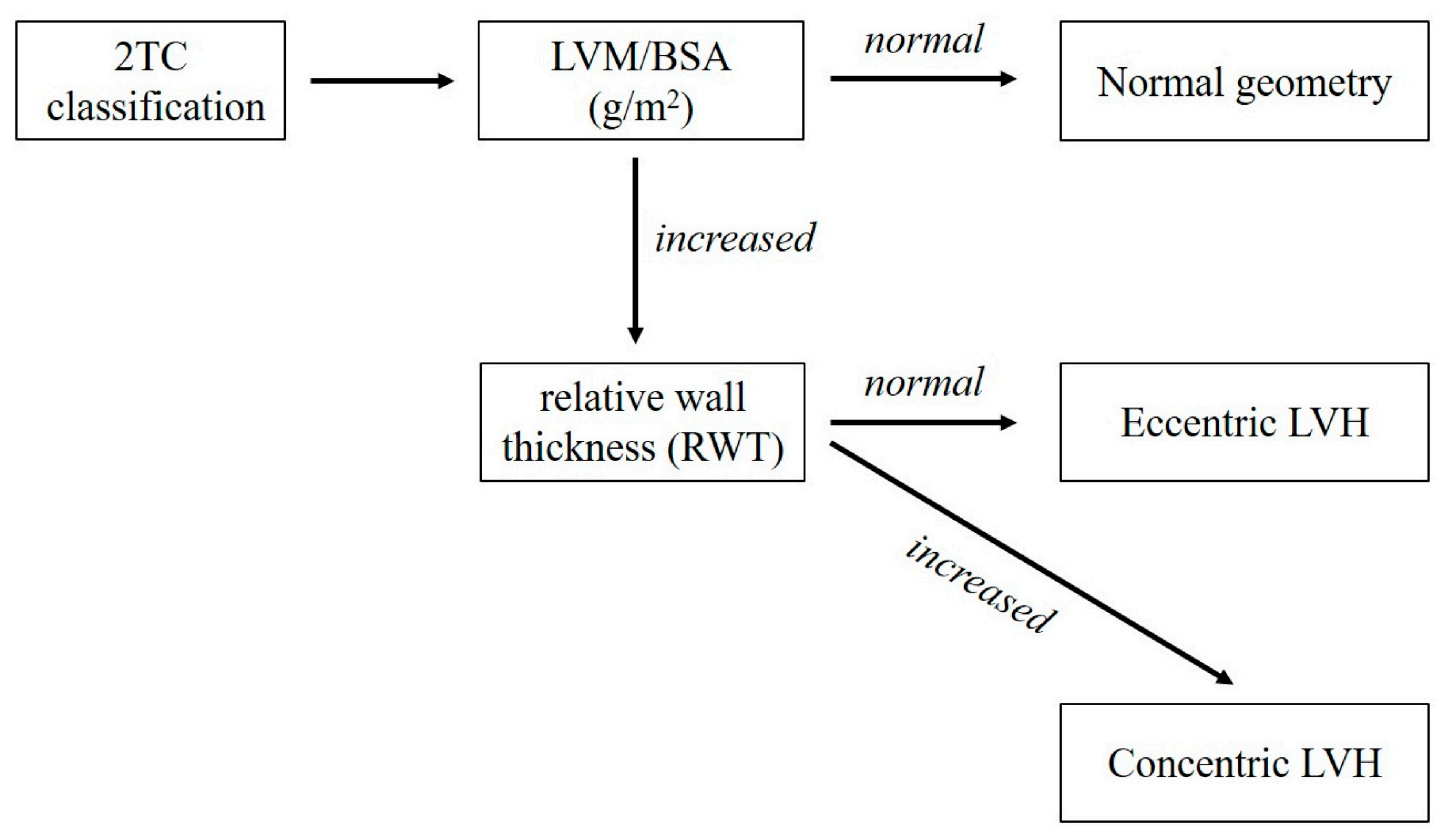
Classification pathway for left ventricular hypertrophy using the two-tiered classification (2TC).

All athletes were classified using the 2TC and 4TC systems. Subsequently, the athletes were grouped according to 2TC and 4TC allocations. We defined LVH in 2TC, in addition to the increased LVM/BSA ratio, as concentric when the relative wall thickness (RWT) increased (≥0.42) and eccentric when concentricity did not increase (RWT < 0.42) (Figure 1, Table S1).

**Figure 2:**
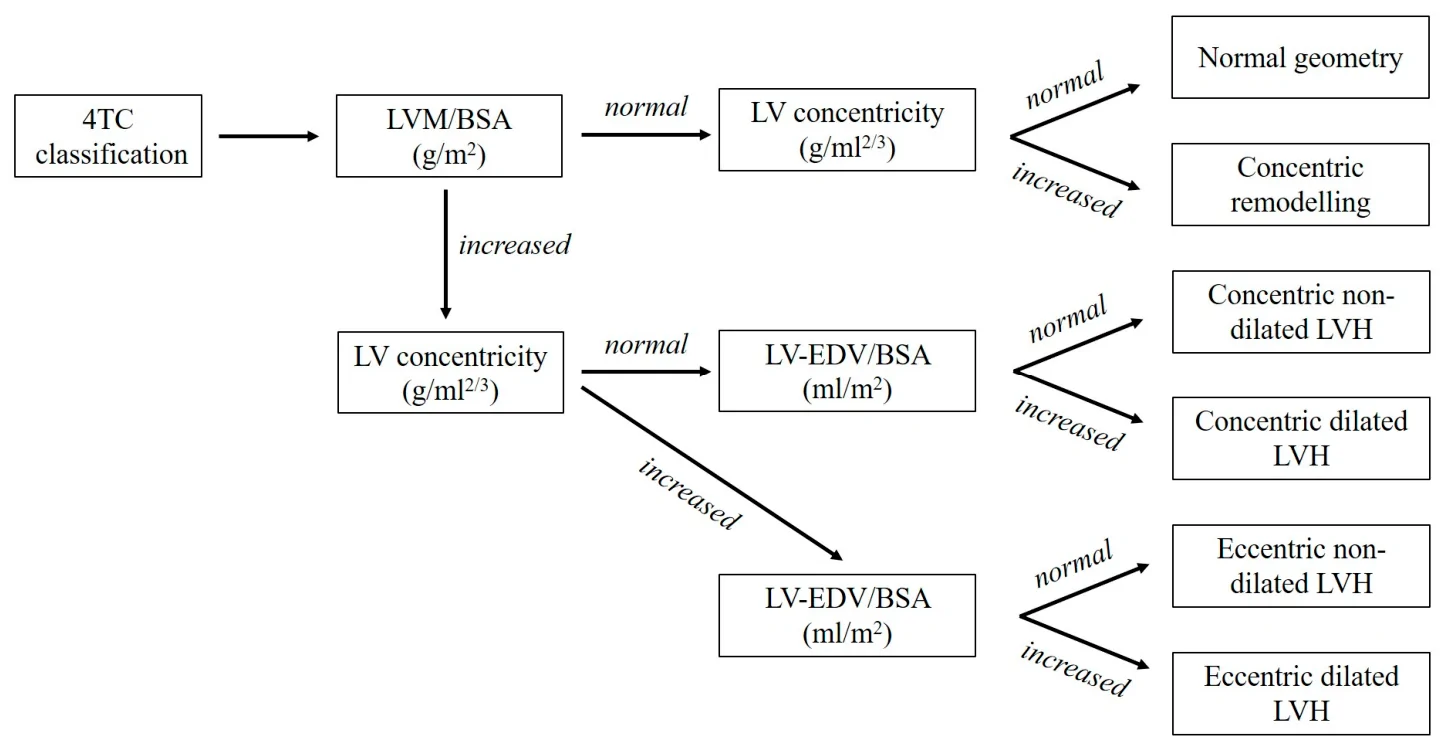
Classification pathway for left ventricular hypertrophy using the four-tiered classification (4TC).

The basis of 4TC was three diagnostic criteria: LVM/BSA, LV-EDV/BSA, and LV concentricity. When neither concentricity, the ratio of LVM and BSA (LVM/BSA), nor the quotient of LV-EDV and BSA (LV-EDV/BSA) increased, normal geometry was observed. If the concentricity is higher, but the ratio of LVM/BSA and LV-EDV/BSA is not, we speak of concentric remodeling. In the case of concentric non-dilated hypertrophy, the concentricity and the ratio of LVM/BSA were higher, but the ratio of LV-EDV/BSA was not. If all three parameters are elevated, concentric dilated hypertrophy is observed. In eccentric non-dilated hypertrophy, the increased ratio of LVM/BSA is accompanied by normal concentricity and a normal LV-EDV/BSA ratio. Finally, in eccentric dilated hypertrophy, increased LVM/BSA and elevated LV-EDV/BSA were accompanied by normal concentricity (Figure 2, Table S2).

### 2.3. Statistical Analysis

Data were analyzed using the SPSS 21 Statistical Package for the Social Sciences (Chicago, IL, USA). The normality of quantitative variables was analyzed using the Shapiro–Wilk test.

Normally distributed variables with homogeneous variances were compared between the 2TC and 4TC groups using univariate ANOVA with Tukey’s post hoc testing. Reclassification between the 2TC and 4TC groups was analyzed using a one-sample *t*-test and Wilcoxon test. ANOVA or the Kruskal–Wallis test with Tukey post hoc or Mann–Whitney test were performed to examine the differences between the results of the different groups. Pearson’s correlation test was used to examine the potential correlations between the different echocardiographic and anthropometric parameters. Multivariate analysis was also performed to detect the additive influencing effect of the different anthropometric parameters and cumulative training time. In all cases, *p* < 0.05 was considered statistically significant.

### 2.4. Ethics Statement

This study was designed in accordance with the principles outlined in the Declaration of Helsinki and was approved by the Scientific and Research Ethics Committee of the Health Science Council, Hungary (ETT TUKEB; approval number: 26914-5/2021/EÜIG). Written informed consent was obtained from all adult participants. For participants younger than 18 years of age, written informed consent was obtained from a parent or legal guardian, and assent was obtained from the participant, when appropriate.

## 3. Results

In our research, 121 adolescent and 114 adult athletes were examined in different sports disciplines, and the most important anthropometric data can be seen below in Table 1, showing the significant differences between the two examined groups.

In the athletes’ ECGs, no pathological alterations were observed other than normal changes applied to cardiac adaptation.

### 3.1. Left Ventricular Geometry in Adolescent Athletes

According to the conventional two-tier classification (2TC), 68 adolescent athletes (56%) were classified as having normal LV geometry, 45 (37%) as having concentric LV hypertrophy (LVH), and eight (7%) as having eccentric LVH.

**Figure 3:**
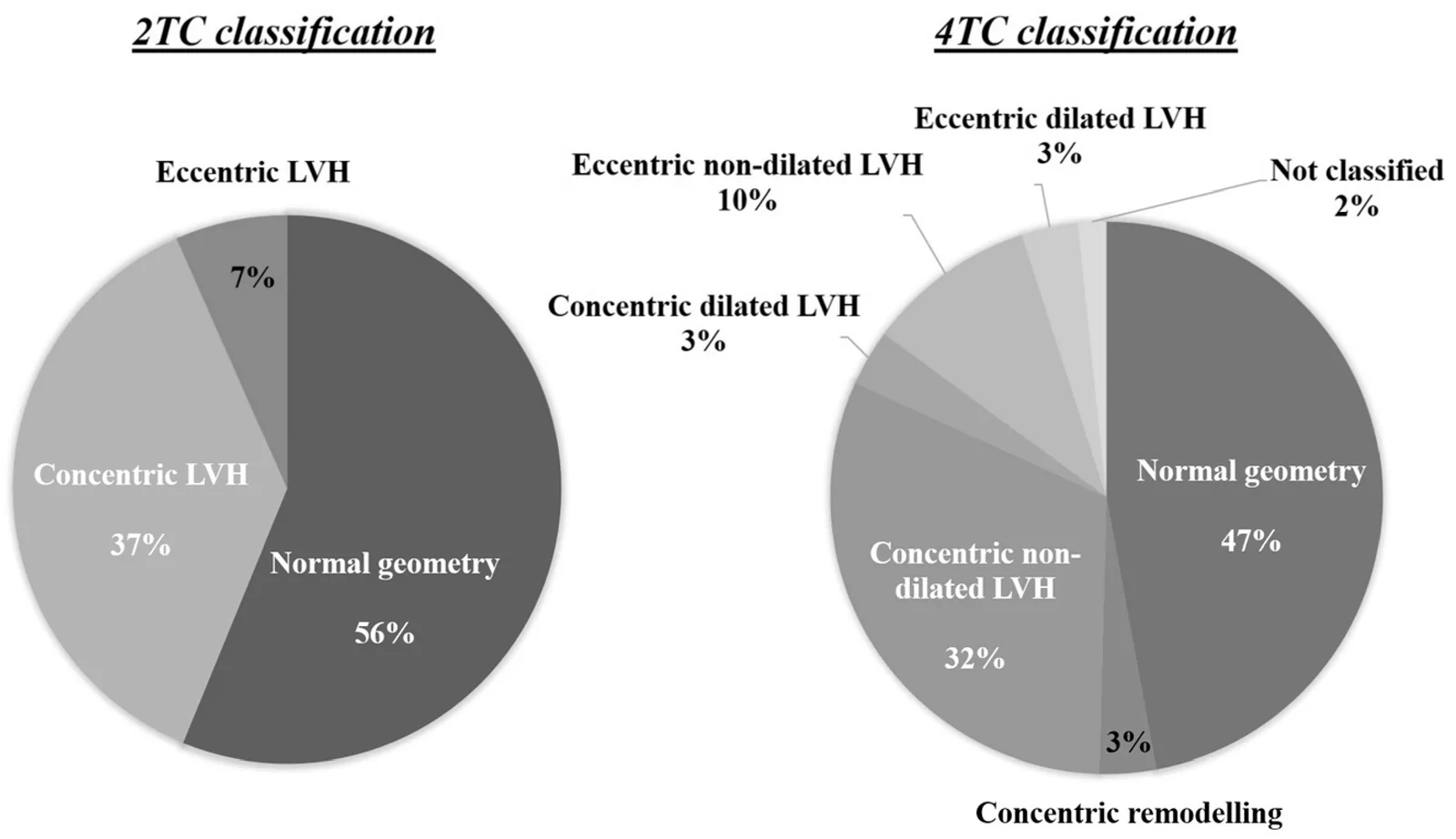
Percentage distribution of the examined adolescent athletes in the 2TC and 4TC allocation.

Using the four-tier classification (4TC), 57 athletes (47%) were categorized as having normal geometry, 38 (32%) as concentric non-dilated LVH, 12 (10%) as eccentric non-dilated LVH, four (3%) as eccentric dilated LVH, four (3%) as concentric remodeling, and four (3%) as concentric dilated LVH. In addition, two athletes (2%) exhibited isolated LV dilatation without evidence of LVH (Figure 3).

Table 2 contains the most important anthropometric and echocardiographic parameters of adolescent athletes examined in detail in the different types of LV hypertrophic groups, also showing the significant differences in the LVH groups’ parameters compared with the normal geometry group.

We did not observe any statistically significant differences in age, LBM, BSA, or cumulative training duration between the normal geometry and LVH groups. Although the cumulative training duration was numerically higher in the concentric dilated LVH group and athletes in the eccentric dilated LVH group were numerically older than those in the normal geometry group, these findings should be interpreted descriptively. Owing to the small sample size of these subgroups (*n* = 4), no inferential statistical tests were performed.

Significant differences in LVM and LVM/BSA were observed in the concentric non-dilated LVH group (*p* < 0.01) for both parameters, and in the eccentric non-dilated LVH groups (*p* = 0.02, *p* = 0.04) compared to the normal geometry group. Moreover, in the concentric non-dilated LVH group, concentricity, IVS, and PWT were significantly higher (*p* < 0.01) than in athletes with normal geometry. In the remaining three LVH groups, numerically higher values were also observed for the classification parameters; however, given the small sample sizes (*n* = 4), these groups were assessed descriptively only. These findings were expected, as these parameters contribute to the definition and allocation of athletes into the respective LV geometry categories within the four-tiered classification.

**Figure 4:**
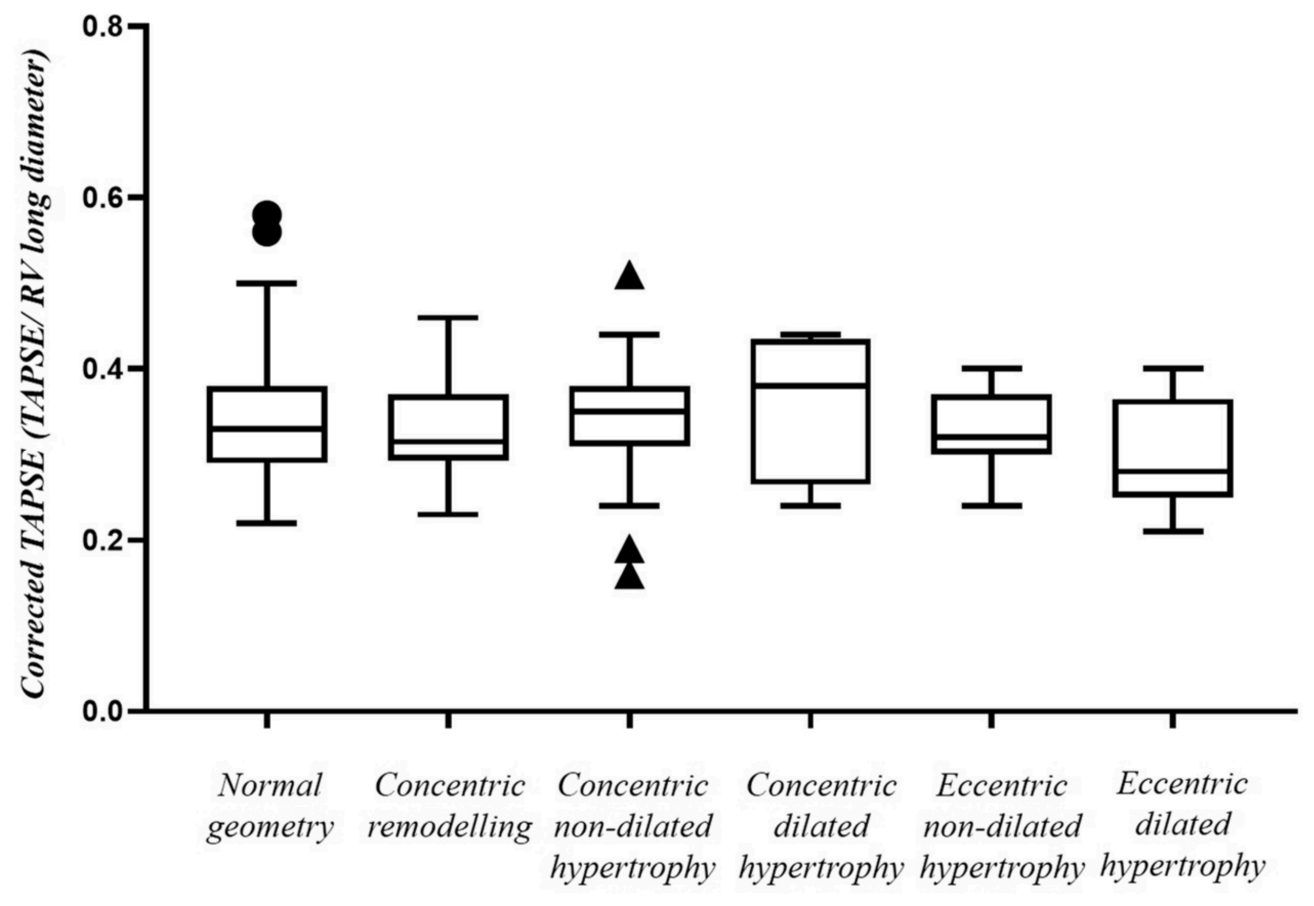
Mean and SD levels of corrected TAPSE in the separate groups. ● and ▲ show outliers. No significant differences were detected between the groups.

**Figure 5:**
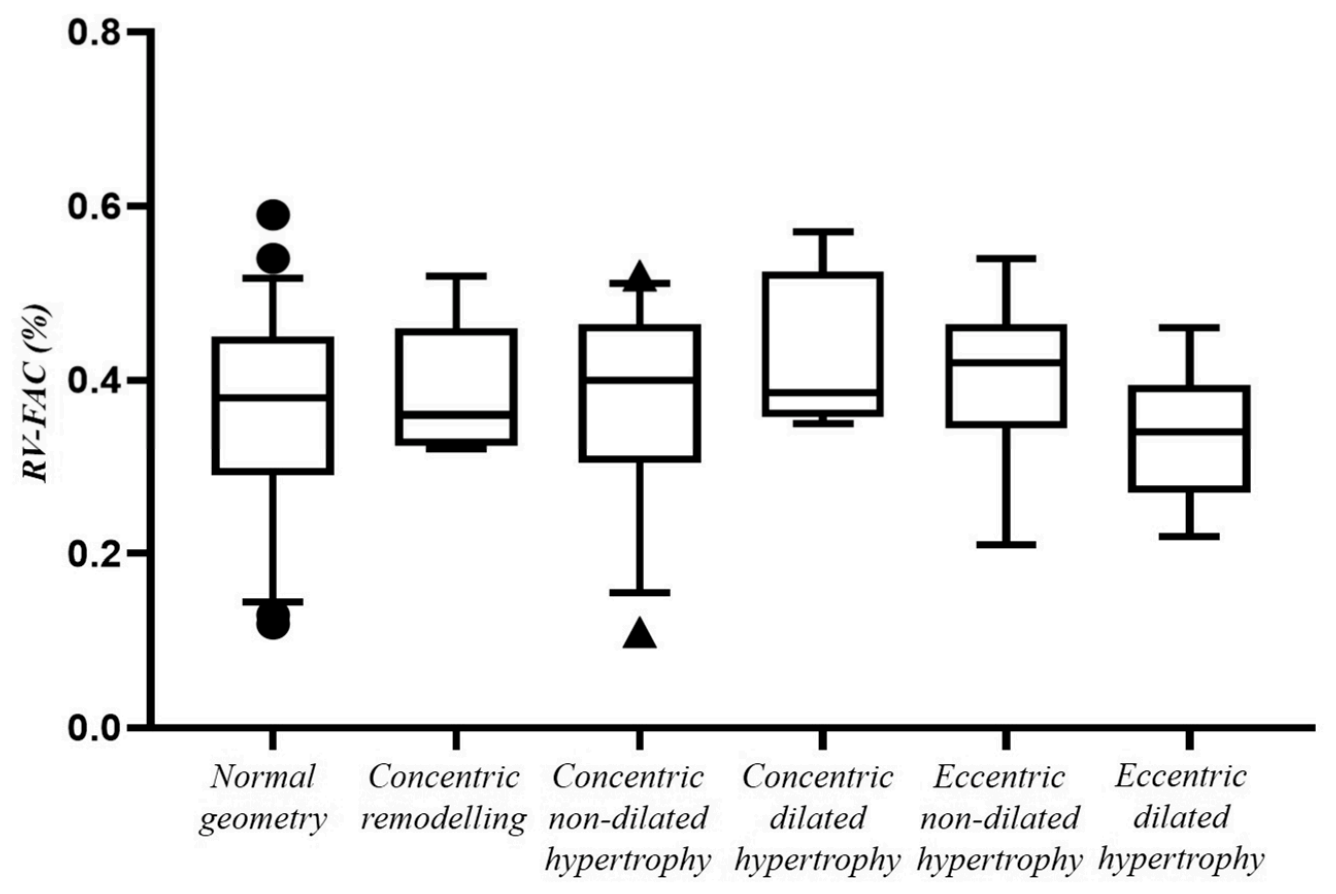
Mean and SD levels of global right ventricular systolic function (RV-FAC%) in the separate groups. ● and ▲ show outliers. No significant differences were detected between the groups.

According to the possible impact of LVH on right ventricular longitudinal (corrected TAPSE) and global systolic function (RV-FAC%), our results clearly showed that there were no significant differences between the normal geometry, concentric remodeling, or any LVH groups (Figure 4 and Figure 5).

Intra- and inter-observer reproducibility was assessed for all echocardiographic parameters, showing good results: the intra-observer ICC was 0.93 (95% CI, 0.88–0.97), while the inter-observer ICC was 0.91 (95% CI, 0.85–0.95).

### 3.2. Correlation Analyses

Different correlation analyses were also performed to examine the connection between the influencing factors (training time, BSA, and LBM) and echocardiographic parameters (septum, PW, LVM, LVM/BSA, LV-EDV/BSA, and concentricity). We detected a significant positive correlation between BSA, LBM, and almost all echocardiographic parameters (except RWT), whereas the training time did not correlate significantly with LV-EDV/BSA (Table 3A). Furthermore, multivariate analysis showed a remarkably significant positive correlation between LVM and the additive effect of cumulative training time, LBM, and BSA (r = 0.785, *p* < 0.001). Examining the connection between LVM and cumulative training time separately in the different LVH subgroups, a significantly weak or medium positive correlation was detected. Correlation analyses showed a weak positive correlation between cumulative training time and LVM/BSA ratio. In the separate LVH groups, no significant correlations were detected between the examined parameters (Table 3B). In contrast, training time with BSA and LBM also showed a remarkable positive correlation (r = 0.443, *p* < 0.001 and r = 0.483, *p* < 0.001, respectively).

### 3.3. Reclassification from 2TC to 4TC in Adolescent Athletes

**Figure 6:**
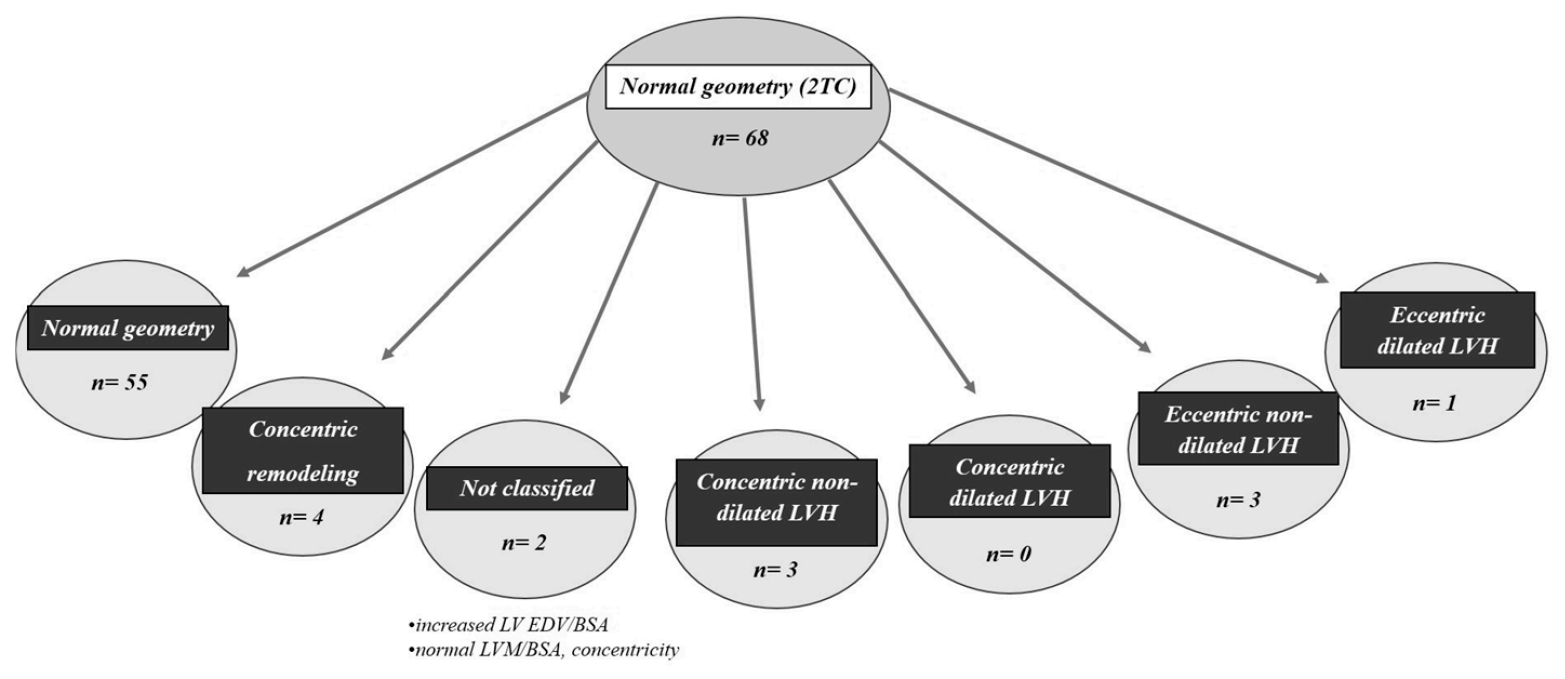
The 4TC allocation of adolescent athletes with “normal geometry” based on the 2TC.

The reclassification analysis demonstrated substantial differences between the two systems. Based on 2TC, 68 patients were categorized as having normal left ventricular geometry. After applying the 4TC allocation, 55 athletes remained in that group. Four individuals were reclassified into the concentric remodeling group, and two could not be classified into any category. Three adolescents were assigned to the concentric non-dilated group and three to the eccentric non-dilated group, respectively. One athlete was reclassified into the eccentric dilated LVH group (Figure 6).

**Figure 7:**
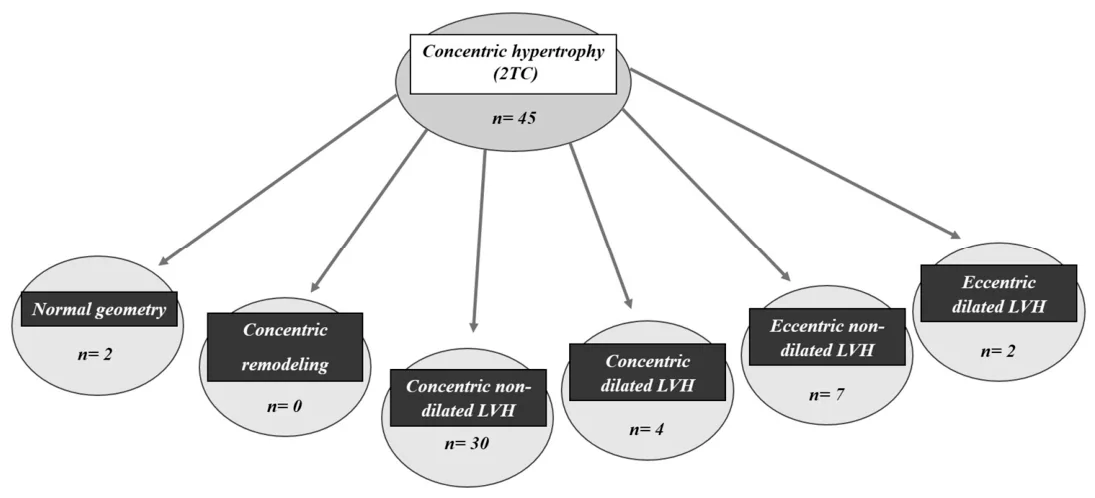
The 4TC allocation of adolescent athletes with “concentric hypertrophy” based on the 2TC.

Figure 7 shows the reclassification of the 45 persons in the category of concentric hypertrophy from the original 2TC into different groups by the 4TC allocation. Two individuals were found to have normal geometry. The non-dilated concentric LVH group included 30 patients. The concentric dilated LVH group included four patients with LVH. Seven athletes were reclassified into the eccentric nondilated LVH category, whereas two athletes were reclassified into the eccentric dilated group (Figure 5).

**Figure 8:**
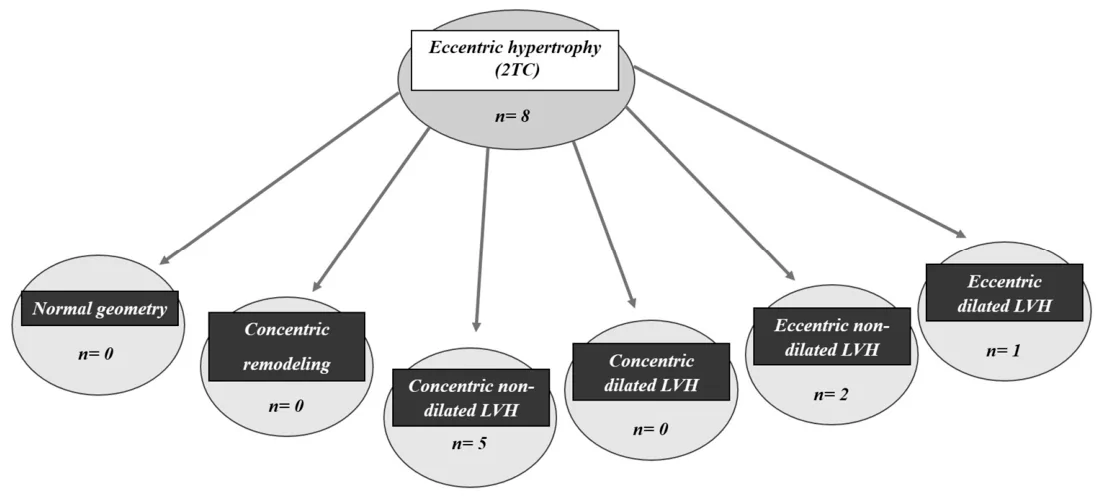
The 4TC allocation of adolescent athletes with “eccentric hypertrophy” based on the 2TC.

Of the eight people originally belonging to the eccentric hypertrophy category, five were repositioned into the concentric non-dilated group, two adolescents into the eccentric non-dilated group, and one into the eccentric dilated subgroup (Figure 8).

### 3.4. Subgroup Analysis According to Sporting Discipline and Sex

Sport-specific descriptive analyses were performed to explore the extent and pattern of reclassification between 2TC and 4TC across sporting disciplines (Table 4).

Among soccer players (*n* = 64) in the conventional 2TC classification, 35 participants (54.7%) were classified in the normal geometry group, 24 athletes (37.5%) in the concentric LVH group, and five players (7.8%) in the eccentric LVH group. The application of the 4TC system revealed considerable heterogeneity within these conventional categories. Of the 35 athletes with normal geometry according to 2TC, 28 (80.0%) retained normal geometry according to 4TC, whereas seven (20.0%) were assigned to the other 4TC phenotypes. Among the 24 athletes with concentric LVH according to 2TC, 19 (79.2%) were classified as having concentric non-dilated LVH, while the remaining athletes were distributed among the other 4TC phenotypes. Notably, of the five athletes classified as having eccentric LVH according to 2TC, only two were categorized as having eccentric non-dilated LVH according to 4TC, whereas three were classified as having concentric non-dilated LVH.

In the runners/ triathletes subgroup (*n* = 22), 2TC identified 15 athletes (68.2%) with normal geometry, six (27.3%) with concentric LVH, and one (4.5%) with eccentric LVH. The 4TC system revealed a substantial redistribution within these categories. Of the 15 athletes with 2TC-normal geometry, 10 (66.7%) retained normal geometry, while the remaining five were distributed across concentric remodeling, concentric non-dilated LVH, eccentric non-dilated LVH, and isolated LV dilatation groups. The six athletes classified as having concentric LVH by 2TC were particularly heterogeneous according to 4TC: one had concentric non-dilated LVH, one had concentric dilated LVH, three had eccentric non-dilated LVH, and one had eccentric dilated LVH. The athlete with eccentric LVH, according to 2TC, was classified as having concentric non-dilated LVH according to 4TC.

In basketball players (*n* = 35), 18 athletes (51.4%) had normal geometry, 15 (42.9%) had concentric LVH, and two (5.7%) had eccentric LVH according to 2TC. Of the 18 athletes with normal geometry, 17 (94.4%) retained this classification according to 4TC, whereas one was classified as having eccentric non-dilated LVH. Among the 15 athletes with concentric LVH, 10 (66.7%) were classified as having concentric non-dilated LVH, two as having concentric dilated LVH, two as having eccentric non-dilated LVH, and one as having normal geometry according to 4TC. Of the two athletes with eccentric LVH according to 2TC, one was classified as concentric non-dilated LVH and the other as eccentric dilated LVH according to 4TC.

Notably, the combined prevalence of eccentric non-dilated and eccentric dilated LVH was descriptively higher among runners and triathletes (22.7%) than among soccer (10.9%) and basketball players (11.5%). Owing to the limited sample sizes of the sport-specific subgroups and the very small number of athletes in several individual geometric categories, reliable formal comparisons of reclassification patterns between sporting disciplines were not feasible. Therefore, the sport-specific analysis was considered descriptive, and no inferential statistical comparisons were performed between disciplines.

Sex-stratified descriptive analysis showed differences in the distribution of LV geometry between female and male athletes. Under the 2TC, normal geometry was observed in 45.8% of female and 58.8% of male athletes, whereas concentric LVH was identified in 45.8% and 35.1%, respectively. Eccentric LVH was uncommon in both sexes (8.4% in females and 6.1% in males). The 4TC classification revealed greater heterogeneity, particularly among female athletes, in whom concentric, eccentric, dilated, and non-dilated phenotypes were identified. Among male athletes, the two largest groups of 4TC were those with normal geometry (48.5%) and those with concentric non-dilated LVH (35.1%). Other LVH categories were relatively uncommon. The sex-stratified analysis also demonstrated that the allocation of athletes under the 2TC and 4TC systems differed in both sexes (Table S3).

### 3.5. Comparison with Adult Athletes

We also examined 114 adult athletes to compare their results with those of adolescent athletes. In the 2TC classification of adult athletes, 60 participants (52.6%) were classified in the normal geometry group, 47 competitors (41.2%) in the concentric LVH group, and the eccentric LVH group included seven athletes (6.2%). In the 4TC allocation, there were 31 subjects in the normal geometry group, 12 in the concentric remodeling group, 49 in the concentric non-dilated group, and two in the concentric dilated group. The eccentric non-dilated group included 14 athletes, and six participants belonged to the eccentric dilated LVH group. The reclassification of adult athletes from the 2TC to the 4TC allocation is summarized in Table 5.

## 4. Discussion

Owing to the increasing number of junior competitive athletes and their early cardiac adaptation, echocardiographic examination of child and adolescent athletes plays an increasingly important role. Left ventricular hypertrophy can be part of normal cardiac adaptation, although previous studies have shown that assessing the type of adaptive left ventricular hypertrophy carries uncertainties depending on the chosen examination method [10,20]. The 4TC of LVH provides a more detailed phenotypic characterization and allows further subdivision of LV geometric patterns. However, in previous studies, 4TC was only administered to adults [10,11]. First, we examined the 4TC classification of adolescent athletes and compared it to their 2TC allocation and that of adult athletes.

Several studies have proven that cardiac adaptation affects athletes at a young age, showing detectable changes, especially in the different echocardiographic left ventricular structural parameters [1,2,21]. In our earlier examination, we also detected significant changes in the LV diastolic and RV structural and functional parameters of adolescent athletes compared to those of age-matched non-athlete controls [22]. In the current study, 53 people (44%) were identified with LVH out of 121 highly trained adolescent athletes, confirming the early cardiac adaptation theory. Applying the 2TC and 4TC in adult athletes, Trachsel et al. proved that concentric hypertrophy can also develop in athletes engaged in endurance training. However, it was also confirmed that applying only 2TC led to a high prevalence of misdiagnosis of LV hypertrophy [12]. Applying the 4TC, the reclassification of athletes occurred from normal to both concentric and eccentric hypertrophic groups, pro and contra. In this study, the application of the 4TC approach resulted in the reclassification of almost 10% of adolescent athletes who were classified as having normal LV geometry according to the conventional 2TC system, demonstrating additional phenotypic differentiation between the two classification approaches. Furthermore, a significant prevalence of redistribution was observed in the different LVH groups. A total of 23 athletes (19%) were reclassified in 4TC into completely different LVH phenotypes.

Based on these results, the 4TC approach provides a more detailed characterization of LVH geometric patterns than the conventional 2TC approach does. Differences in classification between the two approaches have also been reported in elite and young male rugby players, with 4TC providing a more detailed characterization of LV geometric patterns [23,24]. Similarly, a notable redistribution of LVH patterns was observed when the 4TC was applied in non-elite endurance athletes; specifically, 38% of athletes with eccentric LVH according to the 2TC were categorized as having concentric non-dilated LVH according to the 4TC [12]. These previous observations are consistent with our findings, indicating that the application of 4TC results in a different and more detailed characterization of LV geometric patterns. However, the diagnostic or prognostic significance of these differences in classification remains to be established.

In the subgroup analyses according to sex and sporting discipline, reclassification between the 2TC and 4TC approaches was observed across all subgroups, although the extent and patterns of redistribution varied. Notably, among runners and triathletes, the combined prevalence of eccentric non-dilated and eccentric dilated LVH was 22.7% according to the 4TC, compared with 4.5% classified as eccentric LVH according to the conventional 2TC. The higher prevalence of eccentric LVH patterns in this subgroup may reflect the predominantly endurance-based nature and associated hemodynamic demands of these sports disciplines. However, the relatively small subgroup sizes limit the interpretation of sport-specific differences in this study. This finding suggests that the more detailed categorization provided by 4TC may be particularly relevant for the characterization of LV geometry in endurance athletes. Furthermore, the heterogeneity of LV geometric patterns observed within the sports subgroups suggests considerable interindividual variability in exercise-induced cardiac adaptation.

Furthermore, 2% of our adolescent athletes who did not belong to any LVH group presented with increased LV EDV index without any LVH, suggesting that further refinement of the classification may be necessary.

Cumulative training time is one of the most important features in sports. Earlier studies have focused on the possible impact of cumulative training time on the development of LVH. Moreover, not only cumulative training time but also training scope and intensity may influence the type of left and right ventricular adaptation. Bjerring et al. enrolled adolescent competitive and formed endurance athletes in a longitudinal follow-up study. They found that the initial cardiac response was concentric wall thickening due to endurance exercise. Concentric remodeling or hypertrophy appears to be an early step in adaptation. LV dilatation and eccentric hypertrophy can follow sustained high-intensity endurance training in engaged athletes [25]. These findings explain the high percentage of athletes with concentric LVH in our study. Similar findings have been reported in studies on adult athletes. There was no eccentric chamber dilatation until after 6–9 months [26]. In our study, LVM/BSA, LV-EDV/BSA, and concentricity were not associated with training duration. Although the cumulative training duration was numerically higher in the concentric dilated LVH group, this finding should be interpreted with caution because this subgroup comprised only four athletes. No statistically interpretable differences in cumulative training duration were observed across the LV geometry groups, suggesting that the training duration was not clearly associated with LV geometric classification in our cohort. The obvious explanation for this finding is that the enrolled athletes had been playing sports for several years, potentially reducing their ability to detect an association between the training duration and cardiac remodeling. Significant correlations were detected between cumulative training time, BSA, LBM, and the LVM in the entire adolescent group. Moreover, multivariate analysis showed a remarkable additive effect of cumulative training time, BSA, and LBM on LVM values. In contrast, LVM/BSA, LV-EDV/BSA, and concentricity were not correlated with training time. Based on these data, we assumed that training duration may not be the main factor influencing the classification of LVH. The longitudinal study by Weiner et al. confirmed our theory. They enrolled athletes in a longitudinal study for 39 months. They detected important changes in the LV, such as increased LVM and LV-EDV, which appeared after 3 months, and the increment in the following 36 months was less extensive. In the case of LV-EDV, there was practically no significant increase after 3 months [27]. Based on these findings, the type, scope, and intensity of exercise, as well as hereditary factors, probably play a more important role in determining the type of LVH than cumulative training time.

The reclassification in adult athletes showed a pattern similar to that of adolescent athletes. Of the examined athletes, 22% were completely reclassified, and 15% were misdiagnosed as having a “normal heart.” The cumulative training time, LBM, and BSA values were significantly higher in adult athletes than in adolescent athletes in almost all LVH subgroups. Parameters representing the 4TC classification criteria, such as LVM/BSA, LV-EDV/BSA, and concentricity, did not show significant differences between adolescent and adult athletes. These findings suggest that cardiac remodeling develops at a young age, and further cumulative training time has less impact on adaptation. Early adaptation has already been proven in the CHILD study, as well as by Krytofiak et al. [2,21] and Weiner et al. [27]. In contrast, several studies have emphasized the importance of age when high- VO2max training begins. They found that athletes who started training in childhood had eccentric hypertrophy more frequently, whereas athletes who started competitive sports later in life showed concentric hypertrophy patterns [28,29]. Although these studies focused on traditional 2TC parameters, they did not apply 4TC.

In 4TC allocation, we applied sex-specific cut-off values for the LVM index and concentricity to increase the specificity and sensitivity of our examination. Pellicia disserted already in 2012 about the importance of the influence of sex on cardiac adaptation in athletes, explaining the differences between the sexes with different body sizes [26], and the latest sports cardiologic guideline also emphasizes its relevance [30]. Although these recommendations focus on the separate examination of male and female athletes, they do not mention different cut-off values. Krystofiak and co-workers examined child and adolescent athletes and proved that the non-sex-specific LVM normative data—independent of body size (BSA, LBM, height)—are overestimating LVM in boys and underestimating it in girls [31]. Based on this information and the increasing number of women participating in competitive sports, we used different cut-off values for men and women, similar to the methods used in the Dallas Heart Study [9]. In our study, the sex-stratified analysis showed differences in the distribution of LV geometric patterns between female and male athletes. The application of 4TC resulted in a more heterogeneous distribution, particularly among female athletes, with both dilated and non-dilated concentric and eccentric LVH patterns. This observation is particularly relevant in the context of the previously discussed sex-related differences in cardiac dimensions and LV mass and further supports the use of sex-specific cutoff values when characterizing LV geometry in athletes. However, the sex-specific distribution observed in our cohort should be interpreted in the context of the limited number of female athletes included in the study.

The large number of cases of sudden cardiac death caused by idiopathic left ventricular hypertrophy emphasizes the importance of exact LVH classification and screening for maladaptation. A post-mortem examination of athletes who suffered sudden cardiac death proved that idiopathic left ventricular hypertrophy and/or myocardial fibrosis were the cause of death in 10% of those under the age of 18 and in 14% of cases between the ages of 18–35. This ratio increases to 26% in athletes over 35 years of age [9]. Idiopathic LVH is a collective term that encompasses several conditions. From this perspective, it is particularly important to precisely define the type of left ventricular hypertrophy and to assess whether the given hypertrophy type may be compatible with sport-specific demands and the volume and intensity of training. Since we do not have sport-specific normal or cut-off values, it is important to determine the type of left ventricular hypertrophy as broadly and accurately as possible.

We emphasize the need to start sports cardiological examinations as early as possible because cardiac adaptation begins at an early age depending on the sports discipline, age, sex, ethnicity, body size, and possible use of medications or drugs [22,32]. Our findings are consistent with those of previous studies showing that the four-tiered classification provides a more detailed characterization of LVH patterns. Trachsel et al. reported the largest migration from the group of eccentric hypertrophy by the 2TC to the concentric non-dilated group by the 4TC [12]. Our study proved that the diagnosis of LVH can be difficult in adolescent athletes, similar to that in adults. In everyday clinical practice, LVH determination frequently includes only LV wall thickness measurement. However, it is already well known that the sensitivity, specificity, and prognostic accuracy of RWT and wall thickness are low compared to the measurement of LVM, LV-EDV, and their adjusted parameters [31]. Taken together, these findings suggest that the 4TC approach may complement the conventional 2TC system by providing a more detailed characterization of LV geometric patterns, particularly in endurance and high VO_2_max-demanding sports. However, further prospective studies with appropriate reference standards and clinical endpoints are required to establish the clinical and prognostic relevance of these differences.

## 5. Conclusions

The 2TC and 4TC approaches resulted in different assessments of LV geometry in highly trained athletes, with the 4TC providing additional characterization of LVH patterns by considering the presence or absence of LV dilatation. Reclassification patterns varied according to sex and sporting discipline, highlighting the importance of sport-related characteristics when interpreting exercise-induced LV remodeling in athletes. In this context, cumulative training duration alone was not related to LV geometric classification, whereas its combined relationship with BSA and LBM was strongly associated with the LVM. These findings suggest that exercise-induced LV remodeling may be influenced by several factors, including training exposure, body composition, and individual characteristics, rather than training duration alone. The clinical and prognostic relevance of the observed differences in LV classification remains to be established in prospective studies with appropriate reference standards and clinical endpoints in the future.

## 6. Limitations

Our study was a cross-sectional assessment of the prevalence of different types of LVH in adolescent athletes. We consider it necessary to supplement this study with a longitudinal follow-up of the same cohort.

Another limitation is the heterogeneity of the study population regarding the sporting discipline. The athletes represented three different sporting groups: long-distance runners/triathletes, soccer players, and basketball players. Although all disciplines were characterized by a high dynamic component (Group C), they differed in their static component (Groups I–III), which may have contributed to differences in the magnitude and pattern of cardiac adaptation, including LV geometry. Furthermore, the distribution of sporting disciplines differed between the adolescent and adult groups, which may have influenced the observed differences in LV geometry between these age groups.

At the time of this study, new echocardiographic features, such as speckle-tracking strain echocardiography, were not available in our clinic. These examinations would have allowed for a more accurate identification of maladaptive left ventricular hypertrophy and screening of several types of hypertrophic cardiomyopathies.

## Figures and Tables

**Table 1:** The main anthropometric data of the examined adolescent and adult athletes, indicating the significant differences between the two examined groups. Mann–Whitney test, * *p* < 0.05.

	Adolescent Athletes	Adult Athletes
Number of athletes	121	114
Age (years)	15.1 ± 1.6	23.8 ± 3.7
Gender	19.8% girls, 80.2% boys	21.1% women, 78.9% men
Ethnicity	98% Caucasian	99% Caucasian
Height (m)	1.73 ± 0.1	1.78 ± 0.07 *
Weight (kg)	61.36 ± 9.7	71.19 ± 7.6 *
Body surface area (m^2^)	1.73 ± 0.2	1.88 ± 0.2 *
Lean body mass (kg)	50.6 ± 7.2	56.71 ± 8.5 *
Sporting discipline	runners, triathletes: 18.2% (IIIC)	runners, triathletes: 15.8% (IIIC)
	basketball players: 25.6% (IIC)	basketball players: 49.1% (IIC)
	soccer players: 56.2% (IC)	soccer players: 35.1% (IC)
Training volume (h/week)	7.5 h	10.5 h *
Cumulative training duration (h)	2650.5 h	5177.1 h *

**Table 2:** The most important anthropometric and echocardiographic parameters of adolescent athletes examined in detail in the different types of LV hypertrophic groups, also showing the significant differences in the LVH groups’ parameters compared to the normal geometry group. The differences between the different LVH groups and the normal geometry group were examined using the Kruskal–Wallis test and the Mann–Whitney U post hoc test, with Holm correction, * *p* < 0.05, ** *p* < 0.001. Groups with small sample sizes (*n* = 4) were only assessed descriptively, using the median, Q1, Q3, and range values. The shadow and bold formatting were used to highlight statistically significant results.

	Normal Geometry *n* = 57	Concentric Remodeling *n* = 4	Concentric Non-Dilated LVH *n* = 38	Concentric Dilated LVH *n* = 4	Eccentric Non-Dilated LVH *n* = 12	Eccentric Dilated LVH *n* = 4
Age (years)	14.69 (1.51)	14.5 [14–15.75] (4.00)	15.49 (1.19)	16.00 [13.75–16.75] (4.00)	14.56 (1.24)	17.000 [15–17] (4.00)
LBM	48.20 (8.14)	54.50 [51.18–57.0] (10.00)	54.48 (9.19)	47.30 [44.3–55.1] (13.00)	48.20 (7.04)	52.00 [46.5–57.5] (26.60)
BSA (m^2^)	1.66 (0.19)	1.84 [1.74–1.86] (0.25)	1.82 (0.23)	1.68 [1.61–1.85] (0.28)	1.67 (0.16)	1.76 [1.66–1.89] (0.71)
Cumulative training duration (h)	2611.09 (154.60)	2084.00 [1368.75–2799.25] (2167.33)	2810.82 (183.58)	3366.00 [2481.0–4251.5] (2461.36)	2406.00 (368.84)	2200.00 [1584.25–2815.75] (1907.14)
LVM	149.33 (36.57)	197.00 [189.5–205.5] (21.00)	**231.11** (41.41) **	226.50 [202.0–260.75] (78.00)	**181.89** (34.73) *	200.00 [188.00–242.00] (132)
LVM/BSA (g/m^2^)	88.94 (15.53)	108.40 [106.68–111.01] (9.11)	**126.44** (12.28) **	135.37 [122.75–143.88] (24.26)	**108.23** (14.04) *	118.99 [112.59–127.39] (31.42)
RWT	0.44 (0.02)	0.43 [0.38–0.45] (0.11)	**0.49** (0.05) *	0.48 [0.45–0.49] (0.20)	0.45 (0.06)	0.47 [0.39–0.48] (0.13)
LV-EDV/BSA (mL/m^2^)	59.49 (9.33)	50.36 [49.58–52.49] (16.19)	56.56 (7.06)	74.05 [70.10–77.23] (9.19)	62.77 (6.26)	75.68 [72.99–78.16] (19.96)
Concentricity	6.95 (1.22)	9.67 [9.37–10.05] (2.07)	**10.52** (1.19) *	8.67 [8.52–9.83] (1.69)	8.12 (0.87)	7.86 [7.63–8.52] (2.52)
IVS (mm)	9.48 (1.27)	10.50 [10.00–11.00] (2.00)	**11.70** (1.19) *	11.50 [10.25–12.0] (2.00)	10.44 (0.34)	11.00 [10.5–11.00] (4.00)
PWT (mm)	9.93 (1.44)	11.00 [10.00–11.00] (1.00)	**12.03** (0.96) *	11.00 [11.25–12.75] (2.00)	10.67 (1.00)	11.00 [10.5–12.00] (4.00)
LV-EF(%)	60.59 (6.23)	58.75 [54.13–65.88] (18.00)	62.25 (4.48)	63.00 [57.75–67.12] (11.50)	60.83 (3.37)	59.00 [57.5–62.5] (12.50)
e’ mean (cm/s)	18.68 (3.00)	19.00 [17.25–21.50] (5.00)	18.12 (2.18)	17.00 [15.88–20.00] (5.50)	19.22 (2.18)	18.00 [16.50–19.75] (6.50)
E/e’ mean	5.69 (1.08)	5.37 [5.00–5.75] (2.29)	5.69 (0.91)	5.53 [ 4.84–6.75] (2.25)	5.22 (0.18)	5.28 [4.99–6.12] (1.24)

**Table 3:** (**A**) Correlation between the most important echocardiographic parameters (septum, PW = posterior wall, RWT = relative wall thickness, LVM = left ventricle mass, LVM/BSA = left ventricle indexed to body surface area, LV-EDV/BSA = left ventricular end-diastolic volume indexed to body surface area, concentricity) and BSA = body surface area, LBM = lean body mass and cumulative training time. (**B**) Correlation between cumulative training time and LVM/BSA in all adolescent athletes and in the different LVH = left ventricular hypertrophy subgroups. Statistical analysis: Pearson correlation analysis. ** *p* < 0.001, * *p* < 0.05.

(**A**)			
	**BSA**	**LBM**	**Cumulative Training Duration**
Septum	r = 0.579 **	r = 0.601 **	r = 0.279 **
PW	r = 0.546 **	r = 0.562 **	r = 0.287 **
RWT	r = 0.112	r = 0.126	r = 0.750
LVM	r = 0.718 **	r = 0.730 **	r = 0.380 **
LVM/BSA	r = 0.439 **	r = 0.489 **	r = 0.283 *
LV-EDV/BSA	r = 0.225 *	r = 0.235 *	r = 0.150
Concentricity	r = 0.477 **	r = 0.487 **	r = 0.232 *
(**B**)			
	**Cumulative Training Time-LVM**		**Cumulative Training Time–LVM/BSA**
All adolescent athletes	r = 0.380 **		r = 0.289 **
Normal geometry	r = 0.454 **		r = 0.408 *
Concentric remodeling	r = 0.764 *		r = 0.071
Concentric non-dilated LVH	r = 0.389 *		r = 0.216
Concentric dilated LVH	r = 0.800 *		r = 0.201
Eccentric non-dilated LVH	r = 0.740 *		r = 0.532
Eccentric dilated LVH	r = 0.614 *		r = 0.378

**Table 4:** Classification and reclassification of left ventricular geometry from the 2TC to the 4TC system according to sporting discipline (soccer players, runners/triathletes, and basketball players).

**4TC/2TC Allocation** **in Adolescent Soccer Players** **(*n* = 64)**	**Normal Geometry** **(Person)**	**Concentric LVH** **(Person)**	**Eccentric LVH** **(Person)**
	**35 (54.7%)**	**24 (37.5%)**	**5 (7.8%)**
Normal geometry (*n* = 29) (45.3%)	28	1	0
Concentric remodeling (*n* = 2) (3.1%)	2	0	0
Concentric non-dilated LVH (*n* = 24) (37.5%)	2	19	3
Concentric dilated LVH (*n* = 1) (1.6%)	0	1	0
Eccentric non-dilated LVH (*n* = 5) (7.8%)	1	2	2
Eccentric dilated LVH (*n* = 2) (3.1%)	1	1	0
Dilated without any LVH (*n* = 1) (1.6%)	1	0	0
**4TC/2TC Allocation** **in Adolescent Runners/Triathletes** **(*n* = 22)**	**Normal Geometry** **(Person)**	**Concentric LVH** **(Person)**	**Eccentric LVH** **(Person)**
	**15 (68.2%)**	**6 (27.3%)**	**1 (4.5%)**
Normal geometry (*n* = 10) (45,5%)	10	0	0
Concentric remodeling (*n* = 2) (9,1%)	2	0	0
Concentric non-dilated LVH (*n* = 3) (13.7%)	1	1	1
Concentric dilated LVH (*n* = 1) (4.5%)	0	1	0
Eccentric non-dilated LVH (*n* = 4) (18.2%)	1	3	0
Eccentric dilated LVH (*n* = 1) (4.5%)	0	1	0
Dilated without any LVH (*n* = 1) (4.5%)	1	0	0
**4TC/2TC Allocation** **in Adolescent Basketball Players, Boys (*n* = 35)**	**Normal Geometry** **(Person)**	**Concentric LVH** **(Person)**	**Eccentric LVH** **(Person)**
	**18 (51.4%)**	**15 (42.9%)**	**2 (5.7%)**
Normal geometry (*n* = 18) (51.4%)	17	1	0
Concentric remodeling (*n* = 0) (0%)	0	0	0
Concentric non-dilated LVH (*n* = 11) (31.4%)	0	10	1
Concentric dilated LVH (*n* = 2) (5.7%)	0	2	0
Eccentric non-dilated LVH (*n* = 3) (8.6%)	1	2	0
Eccentric dilated LVH (*n* = 1) (2.9%)	0	0	1
Dilated without any LVH (*n* = 0) (0%)	0	0	0

**Table 5:** Reclassification of adult athletes from 2TC to 4TC allocation.

4TC/2TC Allocation	Normal Geometry (Person)	Concentric LVH (Person)	Eccentric LVH (Person)
	60	47	7
Normal geometry	31	0	0
Concentric remodeling	12	0	0
Concentric non-dilated LVH	6	40	3
Concentric dilated LVH	0	2	0
Eccentric non-dilated LVH	11	3	0
Eccentric dilated LVH	0	2	4

## Data Availability

Data available on request from the authors.

## Supplementary Materials

The following supporting information can be downloaded at https://www.mdpi.com/article/10.3390/jcm15176879/s1. Table S1. Diagnostic criteria for the 2-tiered classification (2TC) of left ventricular hypertrophy. Table S2. Diagnostic criteria for the 4-tiered classification (4TC) of left ventricular hypertrophy. Table S3. Sex-specific classification and reclassification of left ventricular geometry from the 2-tiered classification to the 4-tiered classification system.

## Abbreviations

The following abbreviations are used in this manuscript:

2TC	two-tiered classification
4TC	four-tiered classification
BMI	body mass index
BSA	body surface area
ECG	electrocardiography
IVS	interventricular septum
LBM	lean body mass
LV	left ventricle
LV-EDD	left ventricular end-diastolic diameter
LV-EDV	left ventricular end-diastolic volume
LV-ESD	left ventricular end-systolic diameter
LV-ESV	left ventricular end-systolic volume
LVH	left ventricular hypertrophy
LVM	left ventricular mass
PWT	posterior wall thickness
RWT	relative wall thickness
SCD	sudden cardiac death
