# Supplementary material for: Benefits of Four-Tiered Classification of Exercise-Induced Left Ventricular Hypertrophy in Adolescent Athletes
Source: J Clin Med. 2026 Sep 5;15(17):6879. doi: 10.3390/jcm15176879 (PMC13566173; doi:10.3390/jcm15176879)
Supplement: Supplementary file 1 [file jcm-15-06879-s001.zip › jcm-4481145-supplementary.pdf]

Table S1. Diagnostic criteria for the 2-tiered classification (2TC) of left ventricular hypertrophy.

**2TC - Allocation**	**LVM/BSA (g/m²)** ♂ <112 / ♀ <89	**LVM/BSA (g/m²)** ♂ ≥112 / ♀ ≥89	**relative wall thickness (RWT)** <0.42	**relative wall thickness (RWT)** ≥0.42
Normal geometry	●		●	
	●			●
Concentric LVH		●		●
Eccentric LVH		●	●	

Table S2. Diagnostic criteria for the 4-tiered classification (4TC) of left ventricular hypertrophy.

**4TC - Allocation**	**LVM/BSA (g/m²)** ♂ <112 / ♀ <89	**LVM/BSA (g/m²)** ♂ ≥112 / ♀ ≥89	**LV-EDV/BSA (ml/m²)** ♂ <74 / ♀ <68	**LV-EDV/BSA (ml/m²)** ♂ ≥74 / ♀ ≥68	**LV concentricity (g/ml^(2/3))** ♂ <9.1 / ♀ <8.1	**LV concentricity (g/ml^(2/3))** ♂ ≥9.1 / ♀ ≥8.1
Normal geometry	●		●		●	
Concentric remodeling	●		●			●
Concentric non-dilated LVH		●	●			●
Concentric dilated LVH		●		●		●
Eccentric non-dilated LVH		●	●		●	
Eccentric dilated LVH		●		●	●	

Table S3. Sex-specific classification and reclassification of left ventricular geometry from the 2-tiered classification to the 4-tiered classification system.

4TC/ 2TC allocation in female athletes (n=24)	***Normal geometry*** *(person)* 11 (45.8%)	***Concentric LVH*** *(person)* 11 (45.8%)	***Eccentric LVH*** *(person)* 2 (8.4%)
Normal geometry (n=10) (41.7%)	10	0	0
Concentric remodeling (n=0) (0%)	0	0	0
Concentric non-dilated LVH (n=4) (16.7%)	0	3	1
Concentric dilated LVH (n=3) (12.5%)	0	3	0
Eccentric non-dilated LVH (n=4) (16,7%)	0	4	0
Eccentric dilated-LVH (n=2) (8.2%)	0	1	1
Dilated without any LVH (n=1) (4.2%)	1	0	0
4TC/ 2TC allocation in male athletes (n=97)	***Normal geometry*** *(person)* 57 (58.8%)	***Concentric LVH*** *(person)* 34 (35.1%)	***Eccentric LVH*** *(person)* 6 (6.1%)
Normal geometry (n=47) (48.5%)	10	0	0
Concentric remodeling (n=4) (4.1%)	0	0	0
Concentric non-dilated LVH (n=34) (35.1%)	0	3	1
Concentric dilated LVH (n=1) (1%)	0	3	0
Eccentric non-dilated LVH (n=8) (8.2%)	0	4	0
Eccentric dilated-LVH (n=2) (2.1%)	0	1	1
Dilated without any LVH (n=1) (1%)	1	0	0
